# A Case of Cecal Diverticulitis Complicated by Pseudoaneurysm in the Ileocolic Artery

**DOI:** 10.7759/cureus.25680

**Published:** 2022-06-06

**Authors:** Teruaki Inoue, Takahiro Tanaka

**Affiliations:** 1 Department of Internal Medicine, Fujinomiya City General Hospital, Fujinomiya, JPN; 2 Department of Radiology, Fujinomiya City General Hospital, Fujinomiya, JPN

**Keywords:** ileocolic artery, angiography, interventional radiology, pseudoaneurysm, colonic diverticulitis

## Abstract

Acute colonic diverticulitis is a common gastrointestinal illness. It has several complications, such as perforation, abscess, and fistula formation. In addition, pseudoaneurysm caused by diverticulitis has been reported. We report a case of cecal diverticulitis complicated by pseudoaneurysm in the ileocolic artery.

A 58-year-old Japanese man was referred to our hospital for abdominal pain. Abdominal examination revealed right lower quadrant pain. Computed tomography (CT) scans showed the presence of diverticula and pericolic fat stranding in the cecum. Following this, he was diagnosed with cecal diverticulitis. Despite antibiotic treatment, his abdominal pain and blood test results worsened. On the third hospital day, a CT scan was performed again, revealing a pseudoaneurysm with hematoma in the ileocolic artery. Interventional radiology (IVR) was conducted to treat the pseudoaneurysm. It was embolized with n-butyl-2-cyanoacrylate (NBCA) and lipiodol. After embolization, he had stable hemoglobin. His abdominal pain and blood test results improved.

Pseudoaneurysms have been reported as a rare complication for diverticulitis. When a rupture occurs, it has a high risk of mortality. Early diagnosis and treatment of pseudoaneurysms are essential, and we should consider pseudoaneurysms as a complication of acute colonic diverticulitis.

## Introduction

Acute colonic diverticulitis is a common gastrointestinal illness. It is characterized by inflammation of the colonic diverticulum. In Asian populations, colonic diverticulosis more commonly occurs in the right colon. The main symptoms include abdominal pain, fever, and nausea. It has several complications, such as perforation, abscess, and fistula formation. When such complications occur, surgery may be required. In addition, pseudoaneurysm caused by diverticulitis has been reported [1]. The rupture of pseudoaneurysm can cause life-threatening hemorrhage and warrants early intervention. We report a case of cecal diverticulitis complicated by pseudoaneurysm in the ileocolic artery.

## Case presentation

**Figure 1:**
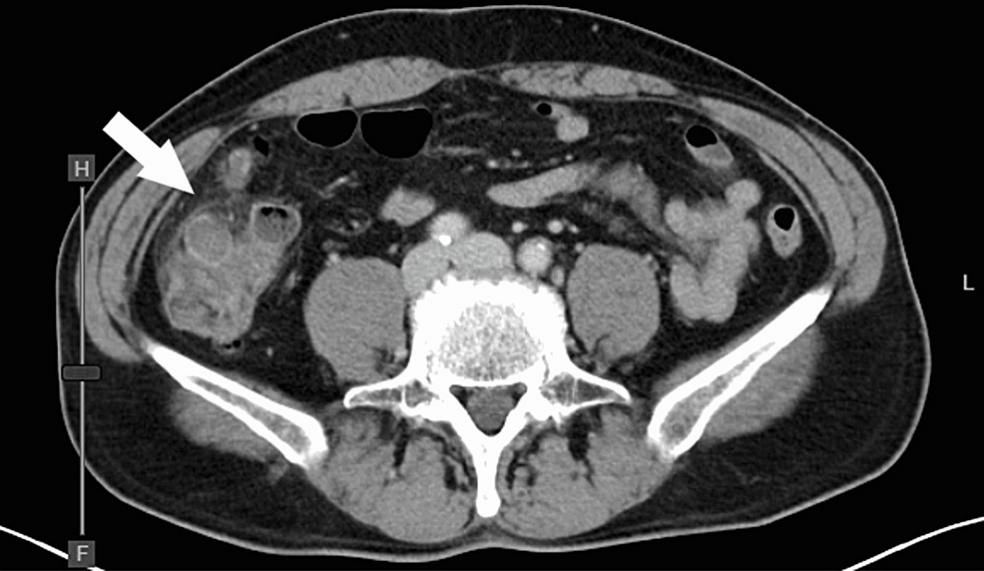
Abdominal contrast-enhanced CT scans, axial view Diverticula and pericolic fat stranding were confirmed in the cecum (arrow).

**Figure 2:**
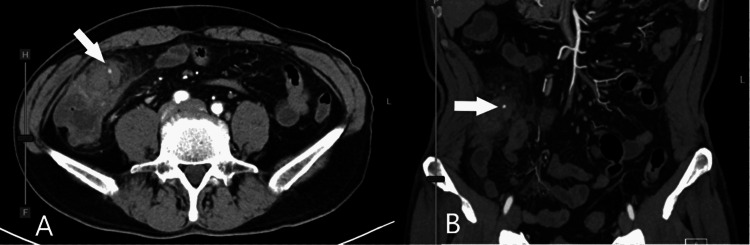
Abdominal contrast-enhanced CT scans in axial view (A) and coronal view (B) Pseudoaneurysm with hematoma was confirmed in the ileocolic artery (arrow).

A 58-year-old Japanese man was referred to our hospital for abdominal pain that was six hours in duration. He had a past medical history of diverticulitis and took no significant medication. Upon examination, he was febrile at 39.2°C, but his other vital signs were normal. Abdominal examination revealed right lower quadrant pain with rebound tenderness. A blood test showed an elevated white blood cell count of 22,200/μL and C-reactive protein (CRP) of 5.67mg/dL. Computed tomography (CT) scans showed the presence of diverticula and pericolic fat stranding in the cecum (Figure 1). Following this, he was diagnosed with cecal diverticulitis and was admitted to our hospital on the same day. He was treated with antibiotics, but abdominal pain worsened, and his CRP level elevated to 32.53mg/dL on a blood test. On the third hospital day, a CT scan was performed again, and it revealed a pseudoaneurysm with hematoma in the ileocolic artery (Figure 2).

**Figure 3:**
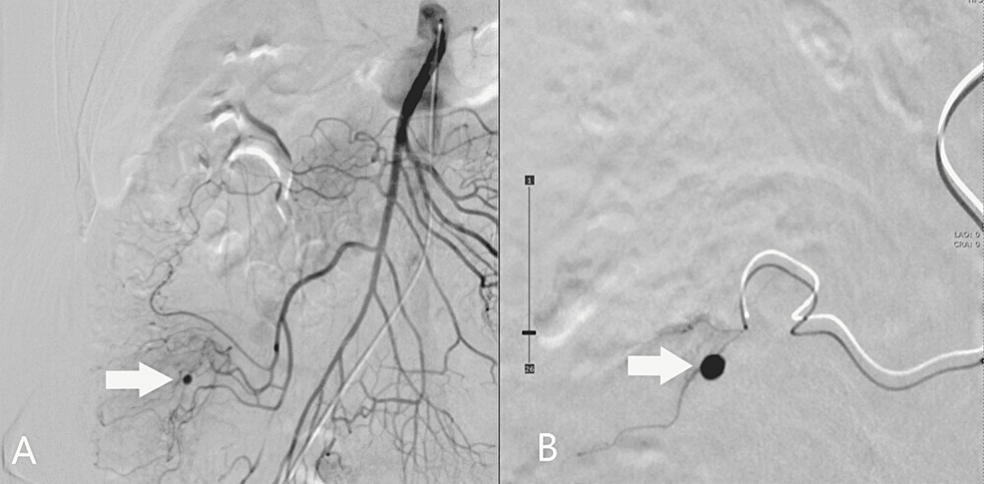
Angiogram Pseudoaneurysm with a diameter of 4 mm was identified in the ileocolic artery (arrow).

**Figure 4:**
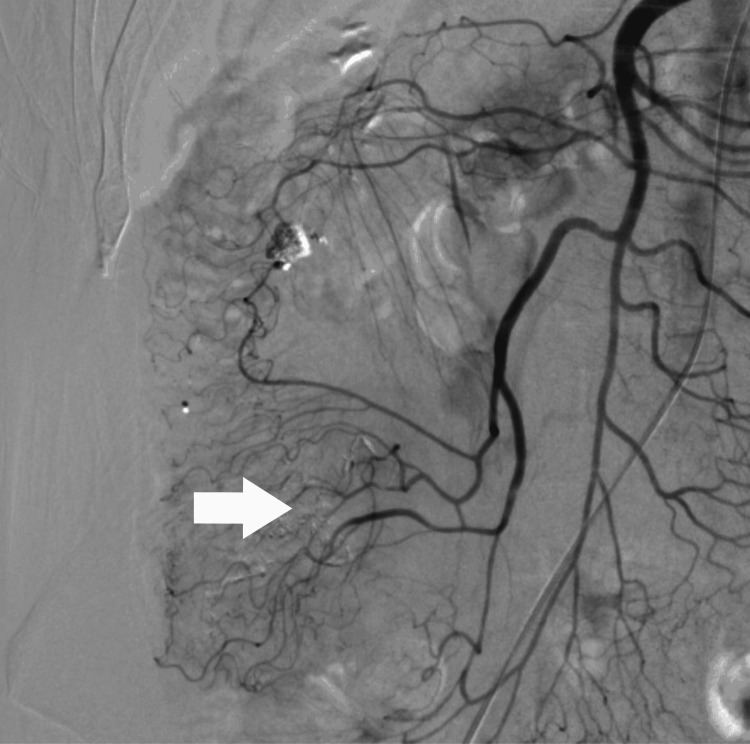
Angiogram Disappearance of the pseudoaneurysm was confirmed (arrow).

**Figure 5:**
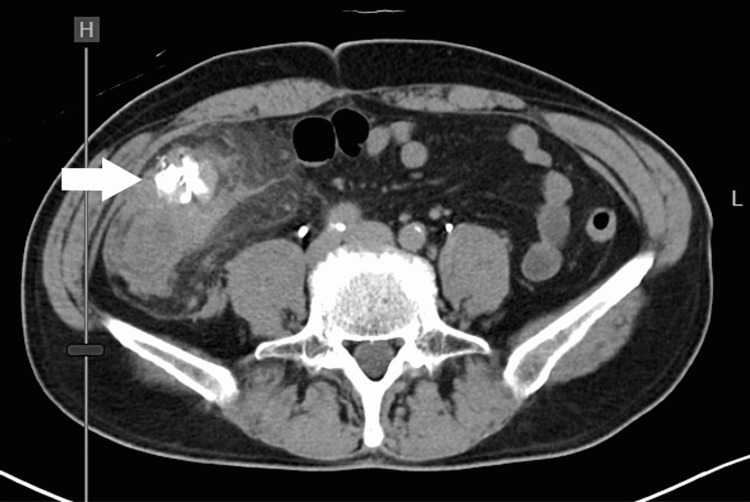
Abdominal plain CT scans, axial view CT scans revealed the presence of lipiodol in the pseudoaneurysm (arrow).

Although the laboratory data showed a normal hemoglobin level and he had no hematochezia which indicates lower gastrointestinal bleeding, there was a risk that massive bleeding occurred due to worsening diverticulitis. Therefore, interventional radiology (IVR) was performed on the fourth hospital day to treat the pseudoaneurysm. Angiography confirmed a pseudoaneurysm with a diameter of 4 mm in the ileocolic artery (Figure 3). It was embolized with n-butyl-2-cyanoacrylate (NBCA) and lipiodol (Figure 4, 5). After embolization, he had hematochezia, but it disappeared in a day. On the seventh hospital day, abdominal pain and blood test results improved, and he started oral intake. He had no recurrent symptoms and anemia. On the 13th hospital day, he was discharged from the hospital.

## Discussion

A colonic diverticulum is a saclike mucosal pouch that protrudes through the muscular layer. Modern studies indicate that the risk of developing diverticulitis is fewer than 5% [2]. Right-sided diverticular disease has a higher incidence of bleeding complications than left-sided [3]. It is attributed to the right colon having diverticula with wider domes and necks [3]. Pseudoaneurysms have been reported as a rare complication for diverticulitis. Janmohamed et al. reported that acute colonic diverticulitis had caused lower gastrointestinal bleeding due to a pseudoaneurysm [4]. Therefore, we should be cautious about pseudoaneurysms in diverticulitis. When a patient with diverticulitis has hematochezia, not only diverticulum bleeding but also pseudoaneurysm rupture should be considered.

A pseudoaneurysm is a hematoma that is formed by a hole in an artery. Visceral artery pseudoaneurysms (VAPAs) are rare, with incidence rates ranging from 0.1 to 2% [5]. In particular, the rate of superior mesenteric artery (SMA) and branches pseudoaneurysm is 5.5 % of all VAPAs [6]. But when the rupture occurs, it has a high rate of mortality (70%) [7]. According to Pitton’s study, the rate of pseudoaneurysm rupture was high at 76.3% [8]; hence, we should treat VAPAs as soon as possible. Delf et al. reported that patients who received anticoagulation with warfarin had a significantly larger pseudoaneurysm [9]. When patients are taking warfarin, we need to be cautious about the development and management of pseudoaneurysms. The causes of pseudoaneurysm are invasive intervention, trauma, and inflammation [10]. In our case, inflammation of diverticulitis is thought to have caused a pseudoaneurysm because the patient had no invasive intervention and trauma. We speculate that the inflammation of diverticulitis destroyed the walls of blood vessels, resulting in pseudoaneurysm formation. For inflammation, there are reports that VAPAs occur in pancreatitis. In addition, Sant’Anna et al. reported a case of ruptured pseudoaneurysm in the ileocolic artery [7].

CT is commonly used to diagnose pseudoaneurysms. However, it does not offer the benefit of treatment, and the sensitivity is 67% [7, 10]. On the other hand, angiography is a reliable technique for pseudoaneurysm diagnosis and enables endovascular embolization [11]. Therefore, angiography should be conducted when the presence of pseudoaneurysm cannot be denied based on CT findings. As in our case, we should suspect the existence of pseudoaneurysm when there is a hematoma around the diverticulum. Additionally, follow-up CT is required when severe inflammation due to diverticulitis continues.

Treatments for pseudoaneurysms include surgery and IVR. Recently, IVR has replaced surgical treatment due to its low morbidity and mortality [12]. Angiography has some advantages for evaluating collateral vessels and accurate localization of pseudoaneurysm. In our case as well, it enabled us to identify the exact localization of the pseudoaneurysm. In addition, compared to IVR, surgery for pseudoaneurysms may require partial or total organ removal. There are several reports of endovascular embolization for pseudoaneurysms [6, 7]. As for endovascular embolization, coils and NBCA have been used. NBCA is useful for embolization. Unlike coils, NBCA is liquid before polymerization, and this makes it possible to treat pseudoaneurysm with multiple outflow vessels [13]. However, it has several complications, such as non-target embolization or catheter trapping, and expertise is required to use it [10].

## Conclusions

In summary, we presented a case of pseudoaneurysm caused by cecal diverticulitis. In our case, early intervention prevented massive bleeding from a pseudoaneurysm. Follow-up CT might be necessary when diverticulitis worsens. In addition, when anemia progresses rapidly, we should be cautious about the complication of pseudoaneurysm in diverticulitis. Early diagnosis and treatment of pseudoaneurysms are essential, and we should consider pseudoaneurysms as a complication of acute colonic diverticulitis.
